# The Use of Radial Artery for CABG: An Update

**DOI:** 10.1155/2021/5528006

**Published:** 2021-04-07

**Authors:** Francesco Nappi, Francesca Bellomo, Pierluigi Nappi, Camilla Chello, Adelaide Iervolino, Massimo Chello, Christophe Acar

**Affiliations:** ^1^Department of Cardiac Surgery, Centre Cardiologique du Nord de Saint-Denis, Paris, France; ^2^Department of Clinical and Experimental Medicine, University of Messina, Italy; ^3^Regenerative Medicine, Università Campus Bio-Medico di Roma, 00128 Rome, Italy; ^4^Department of Cardiovascular Sciences, Fondazione Policlinico Universitario A. Gemelli IRCSS, Italy; ^5^Cardiovascular Surgery, Università Campus Bio-Medico di Roma, 00128 Rome, Italy; ^6^Department of Cardiac Surgery, La Pitié Salpetriere Hospital, Paris, France

## Abstract

We used the radial artery as a second target conduit for coronary artery bypass grafting since 1971. However, randomized clinical studies have demonstrated differences in clinical outcomes between the radial artery and other grafts because these trials are underpowered. As we proceed toward 50 years of experience with radial artery grafting, we examined the literature to define the best second-best target vessel for coronary artery bypass grafting. The literature was reviewed with emphasis, and a large number of randomized controlled trials, propensity-matched observational series, and meta-analyses were identified with a large patient population who received arterial conduit and saphenous vein grafts. The radial artery has been shown to be effective and safe when used as a second target conduit for coronary artery bypass grafting. Results and patency rates were superior to those for saphenous vein grafting. It has also been shown that the radial artery is a safe and effective graft as a third conduit into the territory of the artery right coronary artery. However, there is little evidence based on a few comparable series limiting the use of the gastroepiploic artery. In its fifth decade of use, we can finally deduced that the aorto-to-coronary radial bypass graft is the conduit of choice for coronary operations after the left internal thoracic artery to the left anterior descending artery.

## 1. Introduction and a Historical Note

**Figure 1:**
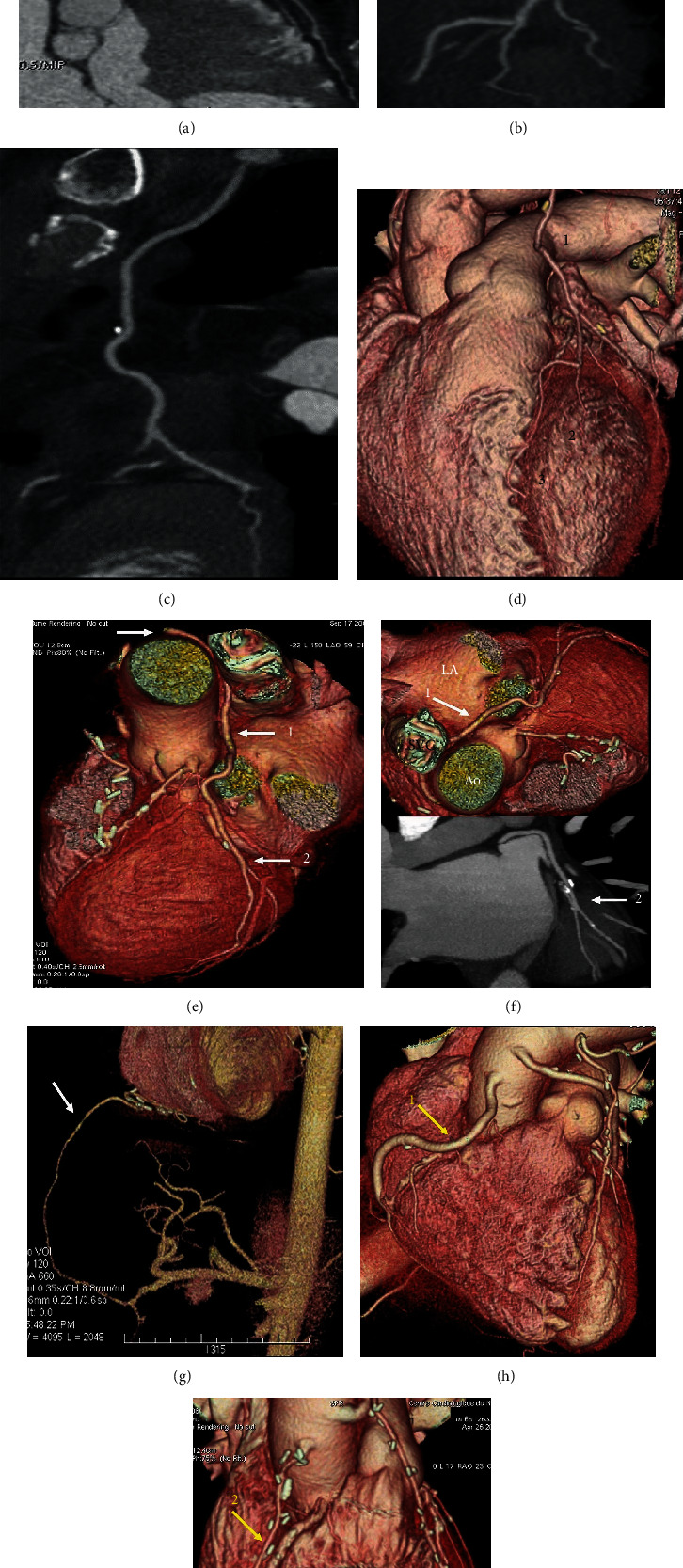
(a–i) Postprocessing of CT angiography of CABG by mean of volume rendering and 2D curved imaging with automatic tracking. (a–c) show 2D curved imaging of CABG with automatic tracking. (a) LITA anastomosed on LAD. (b) LITA sequential grafting on LAD and first diagonal branch. (c) RITA anastomosed on first obtuse branch. (d-i) Volume rendering imaging of CABG. (d) 1-LITA grafted on 2-Diag branch and 3-LAD. (e, f) RITA grafted on CCA. White arrow (1) RITA runs between the aorta and LA, (2) distal grafting on first obtuse branch. (g–i) Comparison between second target conduit on RCA. (g) White arrow gastroepiploic anastomosed to the PDA. (h, i) Yellow arrow (1) SVG and (2) RAG. Note that the venous graft size is greater than the arterial graft size. CABG: coronary artery bypass grafting; LITA: left internal thoracic artery; LAD: left anterior descending; RITA: right internal artery; CCA: circonflexe coronary artery; LA: left appendix; RCA: right coronary artery; PDA: posterior descending artery; SVG: saphen vein graft; RAG: radial artery graft.

We pioneered the use of the radial artery as a graft in coronary artery bypass surgery (CABG) with a single-center series of 910 radial arteries implanted since 1971 [1–6]. Concerns regarding the long-term effectiveness of saphenous-vein grafts (SVG) in coronary-artery bypass surgery have led to numerous debates regarding the conduit of choice in CABG, including bilateral versus single internal-thoracic-artery grafts (LITA) or the radial artery (RA) [7–15]. Based on the current evidence, the LITA remains the prime conduit for its favorable long-term outcomes especially in patients less than 70 years of age without diabetes, indisputably consolidating its role of first-choice graft. A monolithic statement persists: LITA-to-LAD is the single best, possibly the only, important graft in coronary surgery [7, 16–18]. However, the 2017 and 2018 ESC/ESCTS guidelines for CABG have categorized the RA as Class 1A [19], definitively asserting this conduit as the second target conduit for CABG [19–21] (Figure 1).

Since its first use in 1971, the use of RA as a conduit for CABG endured some opposition, unlike the saphenous vein grafts. One-third of recipients in the first series experienced graft occlusion. This prompted Carpentier to proclaim that RA should no longer be used as a graft until this physiological problem was resolved. He suggested that occlusion of this arterial conduit was due to spasm of the denervated vessel [2]. A favorable evolution occurred in 1989 when Carpentier received an angiographic follow-up of a patient operated 14 years earlier from a referring cardiologist demonstrating perfect patency of RA anastomosed to the LAD with no evidence of graft disease. Five additional angiographic follow-ups of recipients grafted with RA conduits from the early 1970s showed a high patency rate, 13–18 years postoperatively. The preparation technique was revised by harvesting the RA “en bloc” with the satellite veins and administration of an antispasmodic drug (Diltiazem).

In our group, we followed Dr. Carpentier's legacy continuing to adopt RA through forty-nine years of practice and the lessons learned on when not to use this conduit rather than how to use it [8–14].

## 2. When Not to Use the Radial Artery?

Many studies including large cohorts of patients have shown that the RA can safely be removed in all cases [22–26]. The variation of the palmar arches distribution has no impact whatsoever. The Allen test whose unreliability is noteworthy [25–27] should not be used. Its application has unnecessarily deprived many patients of the benefits of the radial artery graft (20–40%) [28, 29]. This however does not signify that the RA is a suitable graft in all situations. Contraindications for using the RA are not infrequent and usually are related to the anatomy of the RA itself rather than the variability of the vascular distribution to the upper limb. Appropriate selection of patients for use of RA and a systematic assessment by the surgeon of the contraindications for its use is critical to ensure positive outcomes and survival benefit in CABG.

Key factors to be considered in determining whether RA is indicated are anatomical variation, calcification of RA, and traumatic injury. In our experience, we have individuated the following contraindications.

### 2.1. Anatomical Difference

Anatomical variations of the RA are rare and usually involve the origin of the vessel from the brachial or axillary artery. An abnormality in its origin or variation in its course does not preclude the RA as a graft; conversely, it requires slight changes in the operative technique which are described below. The size of the RA is almost always perfectly matched with that of the coronary vessels. In exceptional cases especially in females (<0.5%), however, severe hypogenesis of the RA (defined as an RA diameter < 1.5 mm despite spasm release maneuvers) can be encountered and constitutes a contraindication. It is then probably preferable to use a saphenous vein as the internal mammary arteries are usually also undersized.

#### 2.1.1. High Birth

The RA commonly originates from the brachial artery in the elbow region, underneath the brachioradialis muscle. Occasionally (4%) [30], it arises more proximally in the midpart of the arm or in the axillary region. The RA then passes superficially in the forearm and lies anterior to the brachioradialis muscle. In high origin cases, it is preferable not to extend the dissection of the radial conduit above the elbow joint.

#### 2.1.2. High Termination

The RA terminates at the level of the wrist joint into a superficial branch that runs at the anterior aspect of the hand and a deep branch that curves posteriorly to the dorsal aspect of the scaphoid bone which supplies the superficial and deep palmar arches, respectively. Rarely (1%), a high RA termination at the midpart of the forearm is observed [30]. In this situation, the RA can be used for coronary grafting together with its branches naturally arranged in a Y-fashion [31, 32] if the diameter of these branches is large enough. In some cases, the deep branch of the RA follows an aberrant route. It curves externally and passes around the external border of the forearm to run at the dorsal aspect of the wrist.

#### 2.1.3. Calcification of the RA

**Figure 2:**
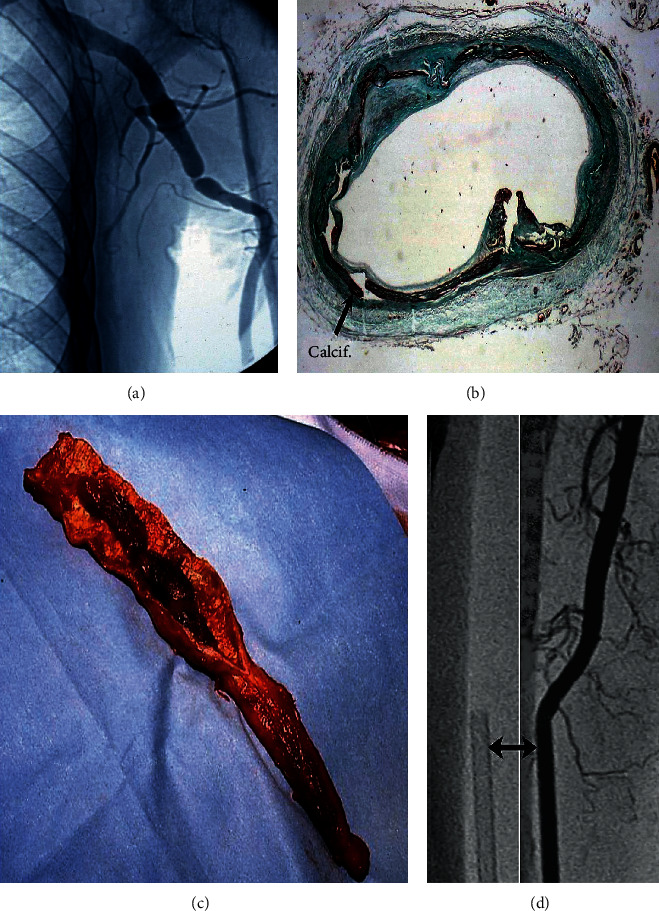
(a) Asymptomatic brachial artery occlusion fortuitously discovered on a postoperative coronary angiogram in a patient having undergone radial artery grafting. This case belongs to the early series. Nowadays, preoperative echo-Doppler of the upper-limb would have revealed the lesion, and RA would not have been used. (b, c) Media calcinosis of the radial artery. (b) Nonobstructive mediacalcinosis in a diabetic patient; (c) atheromatous occlusion with intraluminal thrombus. (d) Traumatic injury for transradial artery coronary intervention. Note that the size of the sheath (arrow) equals that of the vessel lumen.

Atheromatous involvement of the RA is more frequent than the internal mammary artery with a rate of 6% [33] in patients undergoing coronary surgery. These are definite contraindications for RA grafting. However, proximal lesions are not infrequent. When left subclavian artery stenosis is present (up to 2% of patients undergoing coronary surgery) [34], the internal mammary artery flow can be jeopardized, and endovascular stenting may be indicated prior to arterial grafting [34]. In other cases, with proximal upper limb artery obstruction (Figure 2(a)), RA usage is not advised.

In patients with severe diabetes mellitus, the RA can occasionally be the site of medial calcinosis (i.e., nonobstructive calcification of the RA wall) (Figure 2(b)) [35]. The same is true for patients with phosphate/calcium metabolism disorders as in severe renal insufficiency. Furthermore, these patients may require an arteriovenous fistula for chronic hemodialysis; hence, removal of the RA is not recommended. In patients with multifocal atherosclerosis, atheromatous stenosis and occasionally thrombosis of the RA can occur (Figure 2(c)). Angiographic radial artery stenosis was detected in 1.7% of cases in one study [35]. Among patients requiring coronary bypass grafting, the incidence of macroscopic RA calcification is approximately 6% [33, 34]. The ulnar artery is then usually calcified as well. Histological studies have found evidence of microscopic calcification in up to 13% of the cases [36]. Not surprisingly, age as well as classical risk factors for atheroma formation have been identified as determinants of RA calcification [33, 35]. The proximal portion of the RA lying underneath the brachioradialis muscle is less frequently affected by the calcification process [35]. Calcification of the vessel wall, mild or moderate, precludes the use of the RA.

#### 2.1.4. Posttraumatic Radial Artery Injury

The main causes of traumatic injury to the RA are iatrogenic. Focal fibrosis or dissection of the distal portion of the vessel can be observed in patients who have undergone prior arterial punctures either for blood gas sampling or catheterization of the RA for pressure monitoring. Here, the proximal part of the RA is usually spared and can be used for coronary bypass grafting. Conversely, transradial angiography, which is increasingly popular among cardiologists, is a contraindication for RA grafting. Control angiograms have shown a decreased early patency of RA grafts also used for transradial angiography [36]. This approach implies catheterization of the whole conduit with both a guide wire and a coronary catheter. Radial artery spasm frequently occurs, and shearing stress caused by retrograde progression of the material into a collapsed vessel can produce intimal damage [36, 37]. Percutaneous coronary interventions which require insertion of even larger sheaths can provoke devastating injuries with dissection for secondary radial arterial catheterization (Figure 2(d)). Although the vessel usually remains patent, it should not be used for coronary surgery. Fortunately, transradial angiography is most frequently performed at the right forearm, and the RA of the opposite side is then usually available for coronary grafting. The other causes of forearm trauma (wound or bone fractures) very rarely involve the radial artery.

## 3. What to Do When It Fails and How to Prevent It

### 3.1. RA Patency

**Figure 3:**
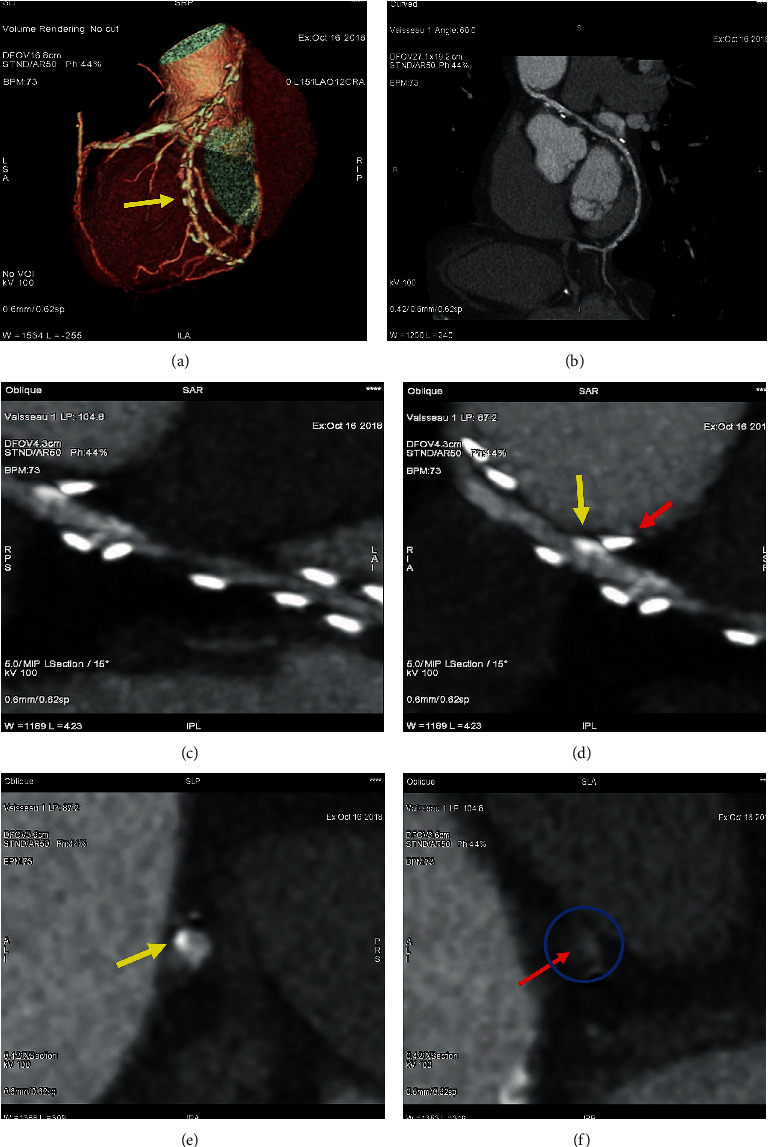
(a–f) CT angiographic control of RA used as second target conduit on second obtuse branch of LCC and PDA. (a) Volume rendering imaging of RA (yellow arrow) running at the level of Theil sinus transversus. (b–f) 2D curved imaging. (b) Course of the radial artery; (c) stenosis of the body of the RA graft. (d) Red arrow shows the metallic clip while yellow arrow highlights a fibrous plaque that are contiguous. (e) Yellow arrow shows a calcific plaque of RA conduit. (f) Note in the blue circle the stenosis of RA due to a fibrous plaque; red arrow shows a remodeling plaque. (LCC2). RA: radial artery; LCC: left coronary circumflex; LCC1: first obtuse branch; LCC2: second obtuse branch; RCA: right coronary artery.

While routine control angiograms were common practice in the early period of RA grafting [4, 7], in the past decade, guidelines have changed our policy for evaluating graft function. Currently, only patients with evidence of myocardial ischemia undergo conventional angiography. The less invasive option of CT angiography can be used in asymptomatic patients and the elderly. It is a good screening tool for assessing graft patency [38, 39], and this method was widely applied to assess long-term RA patency (Figures 3(a)–3(f)) [5]. We reported three distinct angiographic studies to evaluate 1-year, 5-year, and long-term (beyond 5 years and up to 20 years) outcomes [4–6]. We demonstrated that RA patency was 100%, 93%, 83%, and 83% at one month, 1 year, 5 years, and 10 years, respectively [4–6]. RA graft occlusion did not necessarily lead to the return of angina [7]. In fact, we noted that two-thirds of patients with an occluded RA graft persisted clinically asymptomatic in the long term. EKG stress test or scintigraphy frequently is lacking in efficacy to show myocardial ischemia in these patients [5, 6]. Other angiographic studies established that almost all RA grafts remain patent when controlled early in the postoperative period [40, 41] and that some attrition occurred during the first postoperative year (patency: 90-93%) [42, 43]. When compared to our series, similar or slightly higher patency rates have been reported at intermediate-term follow-up: 95%, 89%, and 88% at 4 years [44],5 years [45], and 8 years respectively [8].

In our group [6], all serial angiographic controls collected over 20 years were analyzed. Out of 563 patients, half received at least one coronary angiography. A total of 1427 coronary bypass grafts including 629 RA conduits were evaluated. The angiograms were divided into four uniform arms at different time intervals, and we observed that most RA occlusions occurred within the first 6 months. Evidence showed later than one year radial artery graft patency was significantly stable with virtually no attrition for up to 20 years [6].

### 3.2. String Sign

String sign is defined as a spread narrowing of the entire graft unresponsive to in situ vasodilators. It is normally observed on early surveillance angiograms and concerns approximately 7% of RA grafts within the first postoperative year [42, 46, 47]. Graft involution is likely to result from the competitive flow as shown in several studies, and stress tests might enhance ischemia in the territorial distribution of RAs with string sign [46–48]. Some have suggested that the string sign could be triggered by the use of alpha-adrenergic agents in the perioperative period [47]. Reversal of RA string sign is very rare [49]; these grafts are nonfunctional and should be classified as occluded.

### 3.3. RA Stenosis

Angiographic failure of RA graft occurs more frequently as a complete occlusion and less often a string-like appearance. However, occasionally, focal stenosis of the RA graft has been documented [50]. We reported 6% of RA grafts that were stenosed on subsequent surveillance angiography at 20-year experience [51]. Stenosis was sometimes located at the proximal or distal anastomosis: inadequate surgical technique or intimal hyperplasia could then be implicated. More often, stenosis involved the body of the RA graft (Figures 3(a)–3(d)). Spasm refractory to vasodilators in situ may be difficult to formally rule out. If detected very prematurely in the postoperative time, the stenosis can be handled by balloon dilatation without stenting [50]. However, if discovered later, the stenosis is inclined to manifest organic degeneration. Some narrowing of the RA at the forearm prior to surgery can also be suspected. RA stenosis could also result from an atheromatous plaque that had been overlooked at the time of surgery. As another option, it could be associated to fibrosis secondary to arterial trauma due to poor harvesting technique or to previous catheterization. Indeed, it is not unusual transradial angiography frequently provokes intimal disruption and/or medial dissection [51]. Therefore, RA stenosis occurred secondarily as clearly identified in some controls where the graft was found perfectly intact on a previous angiogram [50]. It is not unlikely that these grafts had been the place of minor parietal lesion that finally evolved into hemodynamic stenosis. Hence, RA graft stenosis can be anticipated by systematic preoperative echo-Doppler screening and exclusion of all calcified RAs as well as previously catheterized conduits. Radial artery graft stenosis can be safely managed with PCI and stents, thus delivering lasting results [50, 52]. The percutaneous procedure for the treatment of vein graft disease continues to be hampered by high periprocedural morbidity resulting from distal embolization of atherothrombotic debris.

## 4. Determinants of RA Patency

### 4.1. Symptoms

Most angiographic studies are directed by symptoms and do not accurately reflect true graft patency. The incidence of graft occlusion is approximately doubled when the indication for angiography is based on evidence of myocardial ischemia [53]. The same is true for the RA graft; whereby, the occlusion rate at 7 years is 12% in asymptomatic patients versus 26% in patients with clinical or electrocardiogram/scintigraphic signs of ischemia [6].

### 4.2. Target Coronary Artery

The target coronary artery is a powerful determinant of patency irrespective of the type of graft whether venous [6] or arterial [54]. Conduits implanted on the left anterior descending coronary artery whose large run-off includes the perforators have the highest patency rate followed by the diagonal and the obtuse marginal branches of the left coronary artery. The right coronary artery has the lowest patency rate due to its territory, mostly limited to the thin right ventricular myocardium [6, 54]. Likewise, the patency of radial artery conduits is directly influenced by the size of the target coronary [46, 47, 53, 55, 56]. RA graft patency for targets on the right coronary artery is statistically inferior to that for targets of the left anterior descending artery. There is also a nonsignificant trend when used in the circumflex artery distribution [57]. In our experience, the number of RA grafts anastomosed to the LAD was too small to allow a proper statistical analysis, but for the other targets, the 7-year patency of RA grafts decreased to 92%, 82%, and 78% for the diagonal, the obtuse marginal, and the right coronary artery, respectively [6].

### 4.3. Competitive Flow

Competitive flow has been recognized as one of the main causes of RA graft failure. Anastomotic patency for targets with moderate stenosis is worse than that for vessels with critical stenosis [4, 45, 56–58]. However, the degree of stenosis labelled as “critical” is a matter of controversy: 70% or 90% according to the authors [59]. In the study by Maniar, the mean degree of stenosis for patent anastomoses was 82% compared with 71% for occluded anastomoses which seems to indicate that the critical range below which flow competition occurs is in between those two values [57].

When combined with valve surgery, the indication for coronary bypass often relies on the detection of coronary stenosis by routine preoperative angiography rather than on the evidence of myocardial ischemia. The risk of grafting a coronary artery bearing a low-grade stenosis resulting in competitive flow is then higher, and the RA graft occlusion rate is increased [56]. Likewise, others have demonstrated that RA graft occlusion occurred more frequently in patients with no/minimal preoperative angina [6].

Coronary bypass for proximal left main stenosis is also at risk for competitive flow. The obtuse marginal RA graft is vulnerable due to unrestricted backflow from the LITA-to-LAD graft towards the circumflex network [60]. Abundant collateral circulation can also compete with the flow from a graft anastomosed to a chronically occluded coronary artery [60].

### 4.4. Sequential Grafting

Sequential grafting is known to improve patency due to the increased distal run-off. Sequential RA conduits have an increased patency compared to grafts with single anastomosis (91% versus 82% at 7 years) [6]. Occasionally, the proximal or the distal end of the graft remains patent whereas the rest of the graft is nonfunctional (string/occlusion) [5, 60].

## 5. RA versus Right Internal Thoracic Artery

The RA provides several benefits over the right internal thoracic artery (RITA); it is an adaptable conduit in which its diameter and length makes it suitable for all coronary targets, even when used for the most distal. It can also be collected at the same time as the LITA, thus reducing operating times. At most, the limitation to the use of the radial artery graft is the possible atheromatous involvement of the vascular structure which is more recurrent than LITA.

The clinical benefits of using RA rather than RITA to integrate IMA to LAD graft from the left were initially evaluated by Borger [61]. It is important to note that patients included in the AR group were older and had a greater number of multiple risk factors such as low ejection fraction, diabetes, and NYHA class; however, a lower incidence of perioperative myocardial infarction than in the RITA group was observed. Evidence therefore demonstrated that the use of the RA as conduit for CABG offered better protection against cardiac death and other cardiac events such as myocardial infarction, readmission, and repeated revascularization, compared to that of RITA conduit during the three-year study period [61]. The limitation of this publication, as of others in the literature, reflects the experience of a single center, and its conclusions remain the subject of debate [62–65]. The use of the radial artery confers the advantage of also reducing wound complications compared to bilateral collection of the ITA, so the selection of this conduit as a second graft to be associated with the LITA remains a valuable option [66–69].

RA and RITA were only compared in the RAPCO (Radial Artery Patency and Clinical Outcomes) randomized trial. The study reported no differences in patency rates between the two conduit and a nonsignificant trend towards better event-free survival for RA at 6-year follow-up [70]. Evidence based on follow-up from retrospective cohort studies addressing the difference between graft patency and composite cardiac endpoints, including rehospitalization rate, is conflicting and usually related to key methodological or sample size limitations [61, 62, 71]. One comparative meta-analysis with clinical endpoints reported comparable mortality; however, a lower incidence of cardiac events, such as myocardial infarction, heart failure, and ischemia, for the RA was noted (RR: 0.49; 95% CI: 0.28 to 0.87; *P* = 0.014) [64]. Moreover, a meta-analysis of comparative network of angiographic studies revealed that the use of RITA conduit resulted in a nonsignificant 27% lowering of absolute risk for late functional graft occlusion compared to RA graft [68]. It should be noted that the results published in a large meta-analysis of 149,902 patients differ, reporting a substantial equivalence in which both conduits (RA and RITA) that were associated with a similar and statistically significant long-term clinical benefit compared to saphen vein [66].

## 6. RA versus Other Grafts

### 6.1. RA versus Vein

In the early postoperative months, graft failure is unlikely to be related to intimal hyperplasia, Instead, immediate graft thrombosis is a recurrent mechanism. As compared to saphenous vein, RA is probably less prone to thrombosis for its superior hemodynamic characteristics [40, 72–74].

Its diameter is only 20% larger than the target vessel allowing a suitable proportions match with the coronary arteries [40]. In addition, this RA is available without valves and its caliber is analogous throughout its progress, with at most a slight decrease in diameter from the proximal to the distal end. Conversely, the diameter of the vein is 50% larger than that of the target coronary artery [40], which often leads to a notable difference. Furthermore, the saphenous vein lumen contains valves and its diameter is inconstant with variation at the level of collateral branches. The diameter of the vein increases from the proximal to the distal end. These relatively adverse hemodynamic features account for a greater thrombosis rate as compared to the RA graft.

Seven RCTs [9–14, 75, 76] have compared the use of RA with saphenous vein graft (SVG) for CABG operation. Extending the follow-up beyond the first postoperative year results in significantly better patency rates for the patients who received the RA compared to that who were managed with the SVG [8]. Two studies reported a lower incidence of clinical events in RA recipients [8]. The randomized trial (Radial Artery Patency Study, RAPS) as well as meta-analyses and observational studies revealed that patients who had the use of radial artery were strongly protected against graft occlusion over 1 year when compared to those who received saphenous vein graft [8, 9, 13, 71, 75, 76]. Radial artery graft occlusion at 1 year occurs in similar proportions in both men and women, whereas, the saphenous vein graft occlusion rate for women is increased twofold when compared to men [77]. Radial artery patency at 1 year is higher in both diabetic and nondiabetic patients compared to vein grafts [8, 78]. A randomized angiographic study one year after total arterial revascularization however failed to detect a difference in graft patency compared to conventional single IMA with veins grafting (Copenhagen Arterial Revascularization Randomized Patency and Outcome trial, CARRPO) [79].

Saphenous veins are affected by an evolving graft disease accountable for increasing intimal hyperplasia, calcification of the vessel wall, and intraluminal congregate of atheromatous debris. At 10 years, only 60% of vein grafts are still patent, and among them, half an atheromasic process occurs in their vessel wall structure [80, 81]. In comparison, 10-year patency rate of RA grafts exceeds 80% in all long-term studies [53, 58] and the RA is virtually free from graft disease [5, 53]. Radial artery versus Saphenous Vein Patency (RSVP) randomized clinical trial revealed that radial artery grafts used on a stenosed branch of the circumflex coronary artery have significantly better patency rate at 5 years than the saphenous vein grafts [9]. Likewise, RA graft patency at 5 years is superior to venous grafts among patients who previously developed in-stent restenosis [78]. Nevertheless, the RAPCO trial which comprised patients >70 years old failed to demonstrate an improved patency in patients who received CABG operation using arterial grafts, with comparable angiographic outcomes at 6-year follow-up, between RA or a vein graft to the largest non-LAD target [82]. Goldman et al. highlighted no differences in death (*P* = 0.61), patency (*P* = 0.82), and composite end point (*P* = 0.31) at 1-year follow-up [14].

Results from a patient-level combined analysis of randomized trials [8] reported a significant interaction between age and the treatment effect on major adverse cardiac events (*P* = 0.04). The age of 75 years was noted as the inflection point; whereby, the radial artery graft is less beneficial. The interaction term analysis revealed a greater benefit in major adverse cardiac event rates with radial-artery grafts than with saphenous-vein grafts in patients younger than 75 years of age (*P* = 0.008), in women (*P* = 0.01), and in patients without renal insufficiency (*P* = 0.02). Diabetes (*P* = 0.35), reduced left ventricular ejection fraction (<35%) (*P* = 0.37), and previous myocardial infarction (*P* = 0.45) did not alter the treatment effect. The target vessel of RA grafting did not significantly affect the treatment effect (*P* = 0.42). Age was an independent predictor of radial-artery-graft occlusion but not saphenous vein graft occlusion. Female sex was associated with a lower risk of radial-artery-graft occlusion and higher risk of saphenous-vein graft occlusion. Long-term use of calcium channel antagonist therapy was combined with a significantly lower risk of radial artery-graft occlusion.

Recently, Royce et al. [15] report a series of 998 unmatched patients at 21-Year survival who received Left Internal Thoracic Artery–Radial Artery anastomosed on a Y graft (*n* = 464) compared to that who were treated with LITA on LAD and SVG. The authors used a 1 : 1 propensity score matching analysis (PMS) to obtain two homogeneous group of 232 pairs. Results revealed an improvement of survival for recipients receiving LITA-RA-Y at up to 21 years (KM, *P* < 0.001) compared to LITA on LAD and SVG as second target vessel for unmatched groups and for PSM groups (KM, *P* = 0.043; HR: 1.3; 95% CI: 1.0 to 1.6; *P* = 0.038). There was no significant difference in survival of the LITA-RA-Y patients compared to other total arterial bypass patients (unmatched and the PSM groups) [15].

## 7. RA versus ITA

Most series reported that the patency rate of the RA graft was lower than that of the LITA graft [3–6, 83–85]. In addition to the type of conduit examined, the interpretation of angiographic data must take into account other determinants of graft failure in order to establish a correct assessment. The patency of the LITA is affected by the size of the target vessel with a lower patency for the right coronary target anastomosis and the highest for grafts placed on the LAD [55]. LITA grafts are also vulnerable to competitive flow with a fourfold increase in graft occlusion rate for vessels with <60% stenosis compared to vessels with 80% stenosis [86, 87]. The patency of the LITA graft was identical to that of the RA grafts at the 5-year follow-up in several studies reporting grafts performed on similar target vessels [3–6, 58, 82].

## 8. RA versus Gastroepiploic Artery

Landmark papers that reported the systematic use of the Gastroepiploic Artery (GEA) conduit for CABG grafting were published by Pym et al. [88] and Suma et al. [89] more than thirty years ago. After initial experiences, the use of GEA grafts have been widely increased. The rate of candidates for a CABG operation who have contraindications to harvesting of GEA is poor. The GEA showed a low incidence of developing severe atherosclerotic degeneration [90] as well as having a good load of flow [91]. In these years, the knowledge on the biological and physiological depiction of the GEA has improved [92], and it is known that the use of this conduit does not increase the rate of postoperative complications [93].

The preferred in situ GEA graft is the distal right coronary artery although the conduit can also be used for grafting on branches of the circumflex artery. It is preferable to graft the GEA in the presence of a subocclusive stenosis (>90%) of the coronary artery in order to maximize the efficacy of flow and avoid a possible failure related to long-standing competitive coronary flow.

Suma et al. reported in patients who received the GEA graft a cumulative patency rate of 97.1% at 1 month, 92.3% at 1 year, 85.5% at 5 years, and 80.9% and 66.5% at 10 years after CABG operation. These not excellent results with evident relatively low patency rates led to a modification of the harvesting technique. Suzuki et al. using skeletonized GEA grafts only for target vessels with stenosis > 90% reported considerably better long-term outcomes (90.2% at 8 years) [94].

Very limited studies are available regarding the safety and effectiveness on the use of GEA compared to the RA second target conduit for coronary artery bypass grafting [67, 95, 96]. Without a doubt, we can say that the usual target of RA and GEA during CABG is the right coronary artery, and they are associated with similar and statistically significant long-term clinical benefits compared to SVG when grafted onto the right coronary artery [66].

However, some points deserve clarifications. First, surgical preference usually dictates the choice of conduit between RA and GEA. Second, it should be noted that a subocclusive (>90%) stenosis of the target coronary artery is crucial for obtaining long-term patency rates. Severe stenosis avoids spasm and eventual failure due to chronic competition of coronary flow. Third, the GEA if often used as an in situ graft at level of the distal RCA [66, 97]. Finally, the use as a conduit for the circumflex system however is limited because of the short graft length compared to the RA, making it a less-attractive option.

## 9. Final Considerations

The consensus on the use of RA as a graft for the second target vessel is not shared unanimously, although the contraindications to the use of RA are scarce. A report from the analysis of STS (Society of Thoracic Surgeons) database revealed that less than 5% of CABG patients received a RA as a second arterial conduit. This trend should be reversed. Randomized clinical trials have worked to facilitate this trend reversal [8, 18, 19] involving many cardiac surgeons in Europe [8, 9, 12] and Australia [10, 11, 15]. If the community has been initially highly reluctant towards RA, the recent evidence promoted a progressive softening of the attitude towards its use as a second arterial conduit.

The results from the current randomized trials on arterial revascularization are still raising contradiction and did not reach a definitive answer on this long-standing debate [7]. The addition of the radial artery as both stand-alone and composite graft (LITA-TA in Y fashion) as shown by Royce et al. [41] is increasingly acquiring more and more consensus [8]. We observed the benefits of RA as a second arterial graft even when it was used in high-risk patients, such as those with reduced ventricular function [98] or unstable angina (freedom from cardiac death at 15 years was 89%) [5, 6]. Furthermore, the location of second RA conduit performed for sequential anastomosis does not impact the late survival benefit, as we demonstrated in our series in 910 patients since 1989 [3, 4] and in more recent reports where 52/57 patients had RA full patency of sequential anastomosis at 9.8 year (91.2% *P* = 0.08) [5, 6].

We have successfully used RA as the first target conduit for coronary artery bypass grafting in patients with myocardial infarction and ischemic mitral regurgitation who had an occluded right coronary artery or an occluded circumflex artery [98–100]. CABG operation with the use of RA was associated with restrictive mitral annuloplastie coupled to papillary muscle approximation in a large percentage of patients [99–102]. In cases of asymmetric tethering with marked distortion of the left ventricle, related by an extensive inferobasal scar, myocardial revascularization using the RA was associated with a restrictive mitral annuloplasty performed using a double row overlapping suture [103].

A dogmatic prejudice on the operative technical challenge together with the influence of the feared financial implications related to sternal wound infection has long limited the use of BITA grafts. It can be perceived that this attitude has been unjustly extending to the RA during the debate on total arterial revascularization, despite the number of favorable evidences published [8]. With over 49 years of experience, we can confidently say that the radial artery is an excellent second option graft for coronary artery bypass surgery [104–107].

Although very long-term evaluation of patients from the early seventies series has revealed that one RA graft by De Oliveira [108] and five RA grafts by Carpentier [1] were fully patent at 23-year follow-up, however, it is indisputable that the choice of the conduit to be used for CABG operation falls within the prerogative of each individual surgeon.
